# Measuring Kinematic Variables in Front Crawl Swimming Using Accelerometers: A Validation Study

**DOI:** 10.3390/s150511363

**Published:** 2015-05-14

**Authors:** Andrew J. Callaway

**Affiliations:** Department of Sport and Physical Activity, Bournemouth University, Poole BH12 5BB, UK; E-Mail: acallaway@bournemouth.ac.uk; Tel.: +44-1202-961255

**Keywords:** biomechanics, swimming, accelerometers, performance analysis, coaching, sports science

## Abstract

Objective data on swimming performance is needed to meet the demands of the swimming coach and athlete. The purpose of this study is to use a multiple inertial measurement units to calculate Lap Time, Velocity, Stroke Count, Stroke Duration, Stroke Rate and Phases of the Stroke (Entry, Pull, Push, Recovery) in front crawl swimming. Using multiple units on the body, an algorithm was developed to calculate the phases of the stroke based on the relative position of the body roll. Twelve swimmers, equipped with these devices on the body, performed fatiguing trials. The calculated factors were compared to the same data derived to video data showing strong positive results for all factors. Four swimmers required individual adaptation to the stroke phase calculation method. The developed algorithm was developed using a search window relative to the body roll (peak/trough). This customization requirement demonstrates that single based devices will not be able to determine these phases of the stroke with sufficient accuracy.

## 1. Introduction

Most swimming research tends to focus on descriptive stroke characteristics, such as stroke rate, because they are more ‘*readily observable*’ [1]. This is equally true when coaching swimming, where coaches will provide extrinsic feedback, involving demonstrations and verbal instructions/descriptions, based on the premise of what the coach could see [2,3]. Previous research has shown this to be bias when considering gross movements [4,5], so when considering detailed biomechanical factors, there will inevitably be a greater degree of inaccuracy.

**Figure 1:**
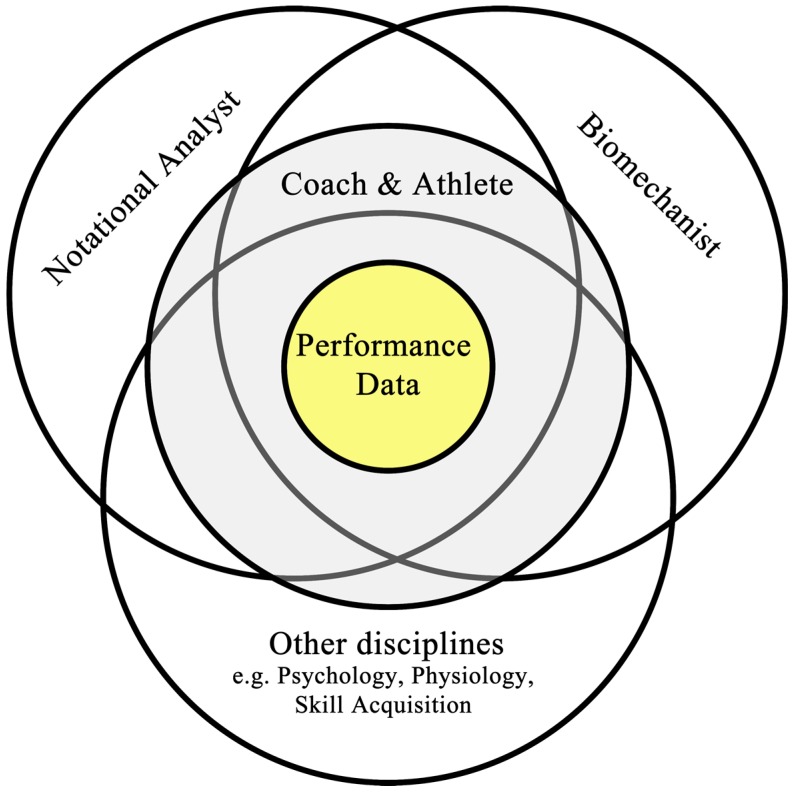
The digital systems data interactions with various other team members. Based on [6].

To overcome this bias, coaches can work with sports science practitioners to employ scientific methods to enhance the level of detail they can use in their coaching practice. Ideally, there will be a coaching team around the coach and athlete/team, which is often only seen at the higher levels of sport. This will allow the practitioner to discuss data with the coach and create extrinsic feedback to help the athlete. The flow of data for this can be seen in Figure 1, developed from Hughes [6]. This shows that the coach will have access to the data from each of the science disciplines to be able to make a decision based on the periodic cycle of the athlete/team, but also, that each scientist has some understanding of their counterparts’ data in order to create a ‘fuller’ picture before making recommendations to the coach. The use of technology in sport can help facilitate this.

Scientific analysis in swimming typically involves either 2D or 3D camera systems [7]. Particularly for coaches, 3D camera systems present with limitations of considerable setup time and cost, which limits their general accessibility [8]. Standard (2D) video technology has become easily accessible due to a general reduction in price, leading to regular use within coaching.

However, there are limitations to the use of 2D methods within swimming, for example, the camera(s) are generally fixed to the pool capturing a limited number of strokes. This can lead to misleading conclusions being drawn on a small sample. Similarly, there is an increasing use of mobile-based technology (e.g., iPad^®^), filming from the side of the pool, above the water limiting the view of underwater actions. An underwater camera can be added to a trolley to move with the swimmer along the pool which would capture a larger dataset of the underwater stroke [9].

**Table 1:** Summary of previous works, with the addition of the contribution from this work. A = Accelerometer, G = Gyroscope, B = Both. ◊ = validity and reliability conducted; * = visually compared to video data.

Author(s)	Number of Sensors	Location(s)	Sensor Type	Lap Time	Velocity	Stroke Rate	Stroke Count	Breathing side	Distance Per Stroke	Stroke Duration	Kick Count	Fatigue	Hand Pitch at Entry	3D Display of Stroke	Upper Body Roll Angle	Lower Body Angle	Body Roll Timing	Body Pitch	Body Roll	Velocity	Stroke Phases	IdC	Symmetry	Body Roll Consistency	Output for Coach
Ohgi *et al.* [10]	1	LW	A										⏍								⏍				
Ohgi *et al.* [11]	1	LW	A										⏍								⏍				
Ohgi and Ichikawa [12]	1	LW	B									⏍	⏍								⏍				
Ichikawa *et al.* [13]	1	LW	B																						
Ichikawa, *et al.* [14]	1	LW	B																						
Davey, Anderson and James [15]	1	LB	A	⏍**◊**		⏍**◊**	⏍**◊**																		
Daukantas *et al.* [16]	1	LB	A			⏍	⏍*																		
Fulton *et al.* [17]	1	Thigh	G								⏍**◊**														
Bächlin *et al.* [18], Bächlin and Tröster [19], Bächlin and Tröster [20]	4	LW, RW, UB, LB	A	⏍	⏍	⏍	⏍		⏍	⏍					⏍*	⏍*	⏍*	⏍*							
Pansiot *et al.* [21]	1	Head	A				⏍	⏍										⏍	⏍						
Nakashima *et al.* [22]	1	LW	B											⏍*											
Le Sage, Justham, Slawson and West [23]	1	LB	B	⏍**◊**		⏍**◊**	⏍**◊**																		⏍
Stamm *et al.* [24]	1	LB	B																	⏍**◊**					
Stamm *et al.* [25]	1	LB	B																				⏍**◊**		
Dadashi *et al.* [26,27]	3	LW, RW, LB	B		⏍**◊**					⏍**◊**											⏍**◊**	⏍**◊**			

There is a plethora of software available (e.g., Kinovea^®^, Quintic^®^ and Dartfish^®^) to aid in the analysis of these videos, some of which are adapted to mobile devices. Whilst these provide substantial functionality for analysis, using them requires a considerable investment of time, especially for a coach or researcher analyzing a swimmer over multiple lengths [23,28].

There are alternatives to video cameras as a means of data capture [29] and with the advances and miniaturization of sensors, these can now often be unobtrusively attached to the body [30] which record data directly and can be processed in ‘*real time*’ to present information to the coach. Wearable sensor technologies hold the key to unlocking novel performance assessment [31] and there is interest in the development of sports technology [32]. Over the last few decades there has been an increase in the use and development of on-body data logger based systems, which use accelerometers and gyroscopes. These tend to offer greater flexibility in data processing methods, in comparison to video based methods, requiring less personnel and technical resources [15]. Generally within swimming however, there is a need for the development of biomechanical testing systems where the timeliness and accuracy of feedback is of ‘*paramount importance*’ [33] (p. 1). With a gradual reduction in sensor size, improved processing power and communication between mobile devices, systems are starting to appear, both in society (e.g., activity monitors) and various sports. However, this does show that biomechanics based equipment is starting to focus on servicing, as opposed to pure research, for which there is a need [34].

Accelerometer based data loggers have been used in swimming to record some factors, which are important to the coach and scientist (see the summary in Table 1). However, these still fall under Glazier’s comment of ‘*readily observable*’ factors. Developing a system, which can identify more detailed factors, such as the phases of the stroke, would allow for additional factors to be calculated such as Index of Coordination [35].

The phases of the stroke have only been observed in the data using accelerometers on the wrists [10,36,37], not fully quantified. Lee *et al.* [38] used a wrist worn sensor with accelerometer and gyroscope on a swimmer on a swim bench, in comparison to video to derive times for the Entry to Entry time (r = 0.97), Exit to Exit time (r = 0.98) and Entry to Exit time (r = 0.85) on a swim bench which lacks some ecological validity, but a great place to start. According to Daukantas, Marozas and Lukosevicius [16], identification of the phases of the stroke using inertial technology is considered feasible, but is a “*long term goal*”.

Furthermore, the potential of the processing ability of the accelerometer based methods have not been extended to measure the individual variations within factors, which could indicate fatigue or technique development over time which could aid both scientists and coaches alike. Rather than coaches ‘seeing’ to make recommendations, we need objective solution to aid their decision-making processes. Burkett and Mellifont [39] (p. 110) agree with this view stating that to meet the:

“...*demands of the swimming coach and athlete, objective data on the swim performance is required*”

The purpose of this study is to collect that objective data by demonstrating the validity and reliability of commercially available accelerometers in their ability to identify the swimmers lap time, velocity, stroke duration, stroke rate and identifying the phases of the stroke. The hypothesis is that a multiple sensor system will be capable of recording all the identified variables in a valid and reliable manner.

## 2. Method 

### 2.1. Participants

With institutional ethical approval, testing was conducted over multiple sessions. Swimmers were recruited from a local swimming club, and volunteered for the testing. The coach of the team withdrew any unfit swimmers prior to testing. Consent forms were completed for the individuals and were supervised by the swimmers’ coaches. The sessions were conducted in a temperature controlled indoor 25 m swimming pool. Data for the system was generated by twelve national level swimmers, comprising of six males (19 ± 2 year, 179.8 ± 13.23 cm, 70.75 ± 15.5 kg) and six female swimmers (17 ± 0.8 year, 165.5 ± 7.04 cm, 54.4 ± 7.9 kg), all injury free, completed the testing protocol after completing informed consent and counter signed by the coach, as their guardian. The swimmers swam 4 × 25 m (100 m) with devices attached at maximal effort, a 8 × 25 m (200 m) maximal effort to induce fatigue (to generate variation within the data) which was not measured, and a final 4 × 25 m (100 m) with devices attached at maximal effort, following a similar procedure to Alberty, Sidney, Pelayo and Toussaint [40].

### 2.2. Experimental Setup

**Figure 2:**
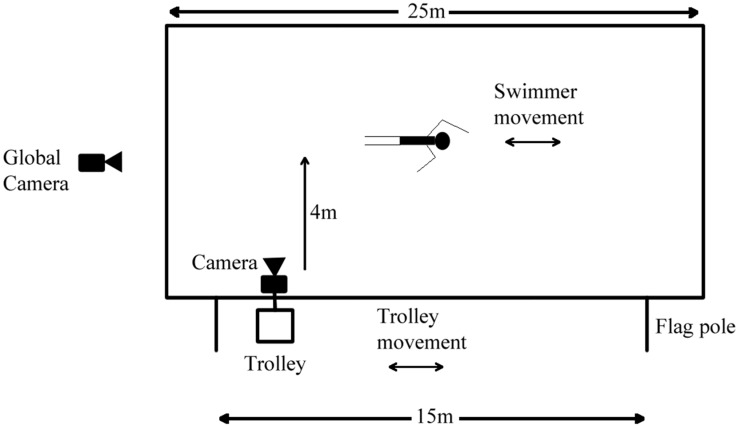
Experimental setup.

Video analysis was used as a validation method with frame rates range from 25 Hz to 72 Hz [9,41,42,43,44,45,46,47,48,49,50,51,52]. An underwater camera (Kodak PlaySport Z × 5, 60 fps) filmed the side view of the swimmer from a trolley [9,51]. A global camera was setup to view the whole experiment and was used as a global time stamp (30 fps). The setup can be seen in Figure 2.

### 2.3. Devices

The waterproof X6-2mini (Gulf Coast Data Concepts, Waveland, MA, USA) is a hermetically sealed, compact tri-axial accelerometer data logger weighing 38 g and measuring 2.95 × 1.6 × 5.9 cm. Each device was set to record at 320 Hz with 16-bit resolution with a full-scale range of ±6 g. Six devices were all were synchronized using the procedures outlined by Callaway and Cobb [53].

Sensors in previous works were placed on “the wrist” [11,12,36,37] and “between the shoulders” [20] and on the “lower back” [24,25]. These do not offer precise, repeatable, anatomical locations for the placement of the sensors. The visual locations shown in these previous works [10,12,20,24,25,36,54] were used as a reference to identify the locations of the sensors in line with ISAK guidelines and using terminology supported by the Australian Sports Commission [55]. The upper arm sensor was placed at the distal end to mirror the placement at the distal end of the forearm for the wrist sensor. This was used to evaluate whether the addition of this device could help with the detection of the phases within the stroke.

**Figure 3:**
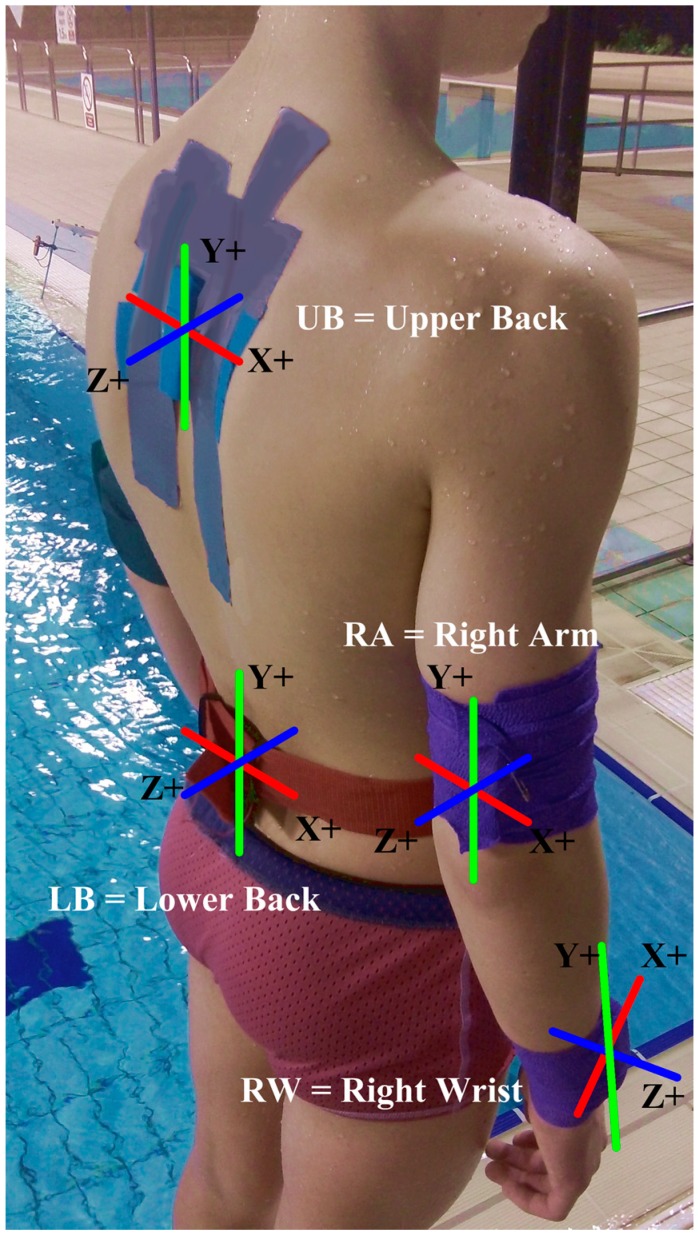
Device placement and attachment method and orientation.

Wrist sensors (LW and RW) are placed at the distal end of the Ulna and Radius, with the -*Y* axis end of the device proximal to the Styloid Process. Arm accelerometers (LA and RA) were placed close to the Olecranon Fossa (elbow). Lower back (LB) sensor was placed at vertebrae L4, in line with the Illiac process. Upper back (UB) was placed on the vertebra T5. These are shown in Figure 3.

The *Z* axis along the Sagittal Plane, *+Z* frontal facing. The *Y* axis along the Coronal Plane, +*Z* frontal facing and the *X* axis along the transverse plane, +*X* frontal facing.

### 2.4. Calculation Methods of Factors Recorded

This section presents the variables derived from the accelerometer system (Table 2). Statistical methods are presented to assess the validity and reliability of the system presented in comparison to video derived data. 

**Table 2:** Variables and calculation method.

Equation	Variable	Calculation Method
1	Lap Time	Wall push off from lower back *y* axis
2	Velocity	$\bar{v}=\frac{d}{t}$
3	Stroke Count	Peak Detection Wrist *x* axis
4	Stroke Duration	$SD=\left(\frac{\sum_{n=1}^{6}\left(LW_{YMin\;n+1}-LW_{YMin\;n}\right)}{5}\right)$
5	Stroke Rate	$SR=60/\left(\frac{\sum_{n=1}^{6}\left(LW_{YMin\;n+1}-LW_{YMin\;n}\right)}{5}\right)$
6	Phases of the Stroke	Wrist Sensors in relation to Body Roll

#### 2.4.1. Lap Time

Verification of the lap times was achieved by taking the lap times viewed from the global positioned camera for 95 laps. Lap times were taken from the videos on two separate occasions, one by the researcher, one by a coach, to ensure inter-rater reliability, which was analyzed using Pearson’s *r* correlation [56]. Results showed a very strong positive correlation between the two video derived lap times (r = 0.977, n = 95, *p* = 0.00), confirming their relative reliability of large effect [57]. Absolute reliability was assessed using the method of mean %error presented by [56]. Maximum and minimum values for the calculation were set to the maximum and minimum values within the data set. The results showed a mean %error of 2.15% (±1.93). The mean of the two inter-rater video derived lap times were then used as a comparison to the lap times from the sensors.

Normality was tested using Kolmogorov-Smirnov test. The lap time data were found to be normally distributed (Video Time from researcher = 0.72, Video Time from coach = 0.524, Mean Video = 0.470, Sensor = 0.711). The mean video lap times were then used to compare to the accelerometer-derived times.

Sensor lap time was calculated using the wall push off seen as a large trough in the data on the *y* axis of the lower back. For each set of 4 laps the swimmer completed, the first wall push off and last wall touch were determined manually clicking on the trough. Between these points, the software automatically determined the push off.

Paired-sample *t*-Test were used for differences between the video times and accelerometer lap times. Average velocity was not assessed using statistics as it is a by-product of a constant (*d* = 25 m) divided by the lap time.

#### 2.4.2. Stroke Count

The number of strokes per lap (each hand entry) for each swimmer were observed from the video and compared to the accelerometer output from the system to check the count. Stroke count was assessed using Pearson’s *r* correlation according the recommendations of O’Donoghue [56].

#### 2.4.3. Stroke Rate and Duration 

The stroke rate and duration were recorded from the video and compared to the accelerometer output from the system. Using SPSS v.19, the data was tested using a *t*-Test to check for significant differences.

#### 2.4.4. Stroke Phases

**Figure 4:**
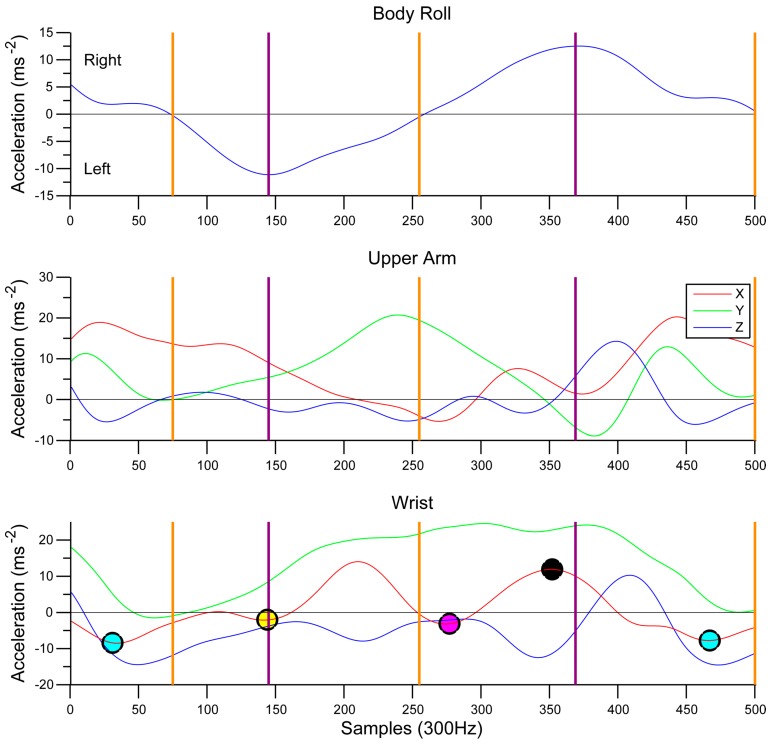
Identification process of the phases in the stroke (Swimmer R). Entry (Cyan dot), Start of Pull (Yellow dot), Start of Push (Magenta dot), Start of Recovery (Black dot). Zero Crossing Body Roll (Orange), Peak Body Roll (Purple).

To develop an algorithm capable of detecting all the phases of the stroke automatically development started with identifying the Hand Entry. This was previously defined as a LW_X_/RW_X_ minima by Ohgi [36,37] which was reconfirmed in the present system using video timings. The algorithm was then developed to find subsequent local minima and maxima using peak detection. These locations were initially identified by taking the time from hand entry, on the underwater camera, and using frame-by-frame analysis method to determine the start point of each phase with a researcher and coach. The time from hand entry of each point could then be plotted against the sensor data to identify the correct minima and maxima of each axis. It was observed that the phases of the stroke coincided with body roll movements allowing further verification of the phases. With the correct maxima and minima detected manually, the algorithm was then designed to automatically find these within 100 sample search windows relative to either a body roll zero crossing (when body level) or peak body roll. Although body roll angle has not been used, the angle can be determined using methods outlined by Bächlin, Förster and Tröster [18]. This method confirms that the zero crossing seen in Figure 4 is when the body is level.

Reliability of the algorithm was analyzed for 78 full strokes of five swimmers. The recovery phase used 71 samples, as on occasion there was no following hand entry to finish that phase for seven of the swimmers viewable on the video camera. The number of swimmers and strokes was selected based on quality of video footage with the best videos of the swimmers chosen offering clear viewing of the swimmer and without the side camera breaching the surface of the water impeding viewing.

Reliability was analyzed using Mean Error (±SD), Mean Absolute Error (MAE), 95th Percentile for MAE, Root Mean Square Error (RMSE), Standard Error from Mean (SEM) for each phase against the durations of the phase from the video [58,59]. Systematic and random bias are also reported for each axis and presented in Bland-Altman plots.

## 3. Results and Discussion

### 3.1. Lap Times

Lap times (n = 95) were derived from video as the wall push off at either end of the pool from the global camera. Correlations between the times from the video and accelerometer show a significant strong positive correlation (r = 0.978, *p* = 0.00). Paired samples *t*-Tests also show that there was no significant difference between the video times (M = 17.24, SD = 1.50) and sensor times (M = 17.28, SD = 1.51) overall; *t*(94) = −1.59, *p* = 0.115.

**Figure 5:**
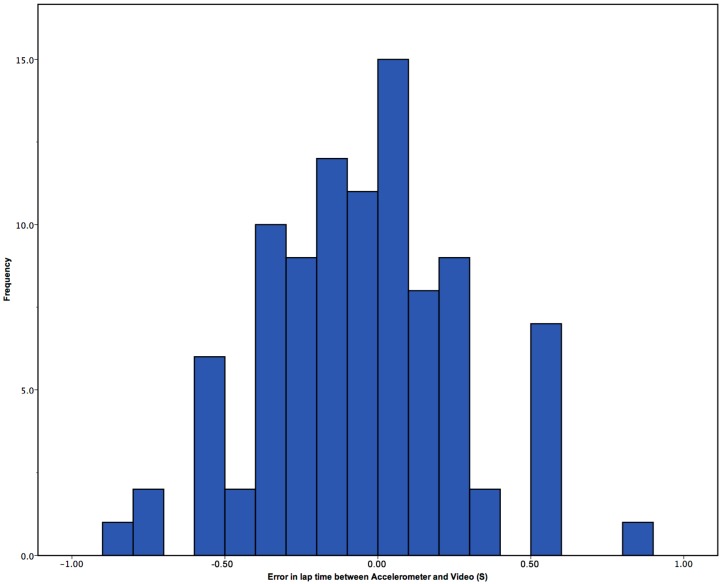
Error in seconds between video and accelerometer for all lap times.

Each swimmer swam four lengths, pre- and post-fatigue. Of these, the start and end of the first and last length was manually selected, not automatically as with previous works [15]. This was due to the nature of the non-wireless devices meaning there was excessive data at the start of a lap, which would not allow for an automatic retrieval of the lap start. It could be that previous works had not full reported that data was trimmed before analysis to allow for the wall push off to be automatically detected. The 2nd and 3rd lap times, from the sensor were automatically detected. Figure 5 shows the frequency of time difference from video the accelerometer in seconds. It shows that generally lap times tend to be recorded as longer using the accelerometer over the video timings. Further analysis was conducted to explore whether there were greater errors in the manual selection method in the sensor data, or automatic detection in the sensor data.

**Figure 6:**
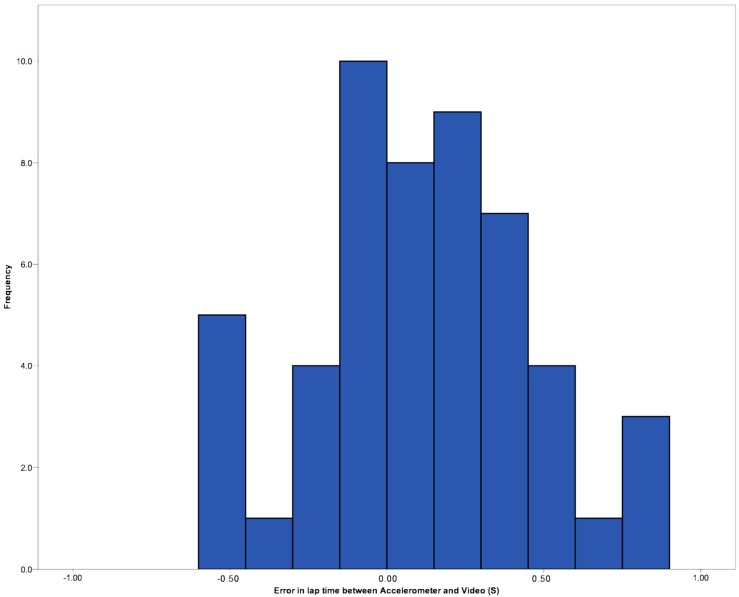
Error in seconds between video and accelerometer manually selected lap times.

With the manual selection of laps there was no significant difference between the video times (M = 16.99, SD = 1.55) and manually selected sensor times (M = 17.06, SD = 1.57) overall; *t*(47) = −1.558, *p* = 0.126. Figure 6 shows the frequency of time difference between video the accelerometer, in seconds, for the manual selection of laps.

It was found that there was some variation in the precision of marker placement placing a marker for the start and end of laps manually. This was due to swimmers having slightly different wall push off techniques. Some dipped under the water and pushed off, others completed a twisting motion where they started almost facing out of the pool and then pushed off facing sideways, gradually rotating before the first stroke. Previous work, such as Davey, Anderson and James [15], used an algorithm to detect the wall push off. Whilst they also found a larger error with the first lap than with the subsequent lap identification, they may have instructed swimmers to all use the same wall push off strategy for this work as there is little identification of any issues to this nature being found. Overall, Davey, Anderson and James [15] did find that the error rates for their accelerometers to videos ranging ±2 s, but more within ±0.5 s. Those results are similar to the present system, however Davey, Anderson and James [15] show a larger range. Davey, Anderson and James [15] identified that lap time variation was due to swimmers finishing the laps in a weak manner, with a soft touch, rather than a competition hard finish. This was also observed with the swimmers in this study, so it would seem that a manual selection method produces a better discrimination of those minor finish peaks, rather than a computational method.

**Figure 7:**
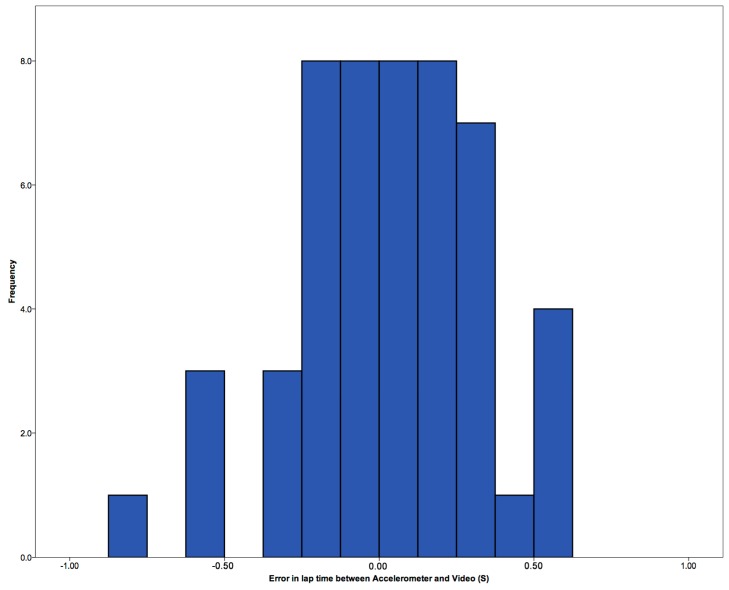
Error in seconds between video and accelerometer automatically selected lap times.

There was also no significant difference between the video times (M = 17.62, SD = 1.44) and automatic lap times from the sensor data (M = 17.65, SD = 1.39) overall; *t*(46) = −0.719, *p* = 0.476 (Figure 7). The results for the middle sections of the laps, the automatic detection, show a higher number within ±0.2 s than with the automatic detection. However, there is one result showing a −1 s result, which appears to be an anomaly. The differences automatic lap time detection could be due to camera position not allowing for a clear observation of the wall push off. This could be developed to include underwater pressure pads as shown by Le Sage, Bindel, Conway, Justham, Slawson and West [23], which could allow for a more exacting lap time to be generated, rather than observations from cameras.

### 3.2. Validation of Stroke Count

**Figure 8:**
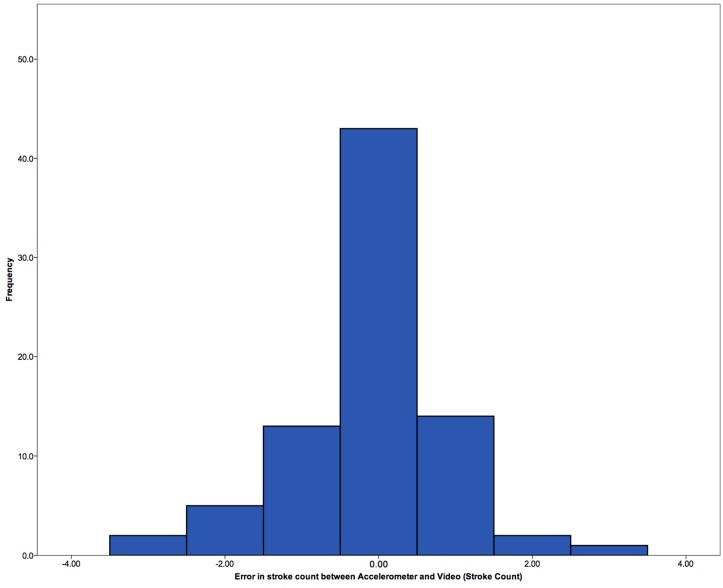
Error in stroke count between video and accelerometer.

Results showed a strong positive correlation (r = 0.948, *p* = 0.00). Davey, Anderson and James [15], using the lower back, are the only authors to validate their stroke count method which was conducted using a difference count. This method was implemented here with results, Figure 8, showing that the majority have no difference, with three results showing up to three strokes difference. Davey, Anderson and James [15] showed limits within ±1 count. This could be because they are using the lower body, compared to upper body in this work. The upper body is filtered to remove any double peaks which can sometimes be seen at the peak, however, on occasion there might be a double peak is found, and records an extra stroke.

### 3.3. Validation of Stroke Rate and Stroke Duration

Validation of stroke rate in previous systems has tended to focus on error reported as a frequency [15,23]. However, considering the type of data, O’Donoghue [56] and Sato *et al.* [60] recommend the use of Correlations. Results show a very strong positive correlation, r = 0.92, *p* = 0.00, (n = 80).

**Figure 9:**
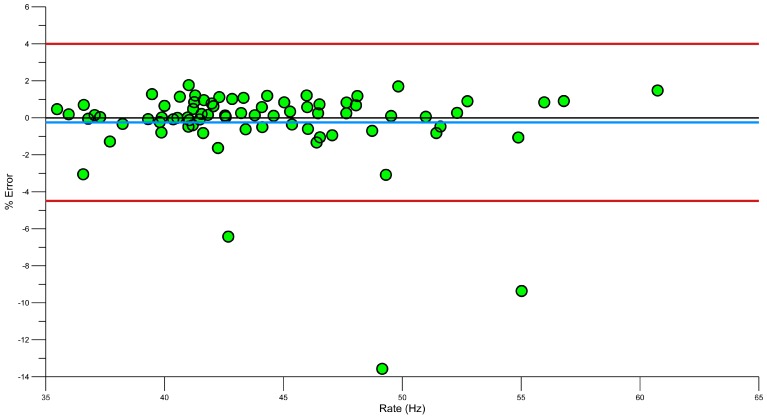
Bland Altman plot showing error in stroke rate (red lines, upper and lower 95% confidence, blue line, systematic bias).

A Bland-Altman plot (Figure 9) shows that there is a mean error of −0.25%, where Le Sage, Bindel, Conway, Justham, Slawson and West [23] showed a similar error of −0.1%. Davey, Anderson and James [15] used six swimmers to generate lap data, which was also recorded manually and on video. They found that the manually calculated stroke rates had a higher error rate than the accelerometer, when compared to those derived by the accelerometer. Using a lower back sensor, the stroke rate error was shown to be between −1 and 2 cycles.

To extend on these previous works, a Bland Altman plot was used to identify any errors in the system. Figure 9 demonstrates the error between video and accelerometer derived stroke rates. The blue line shows the systematic bias −0.25% and a majority of results are within the 95% limits or agreement. Using a regression analysis, some of these errors can be further reduced [61].

Linear Regression analysis produced Equation (1) to allow a correction of the stroke rate:

(1) y=1.274+0.968x

**Figure 10:**
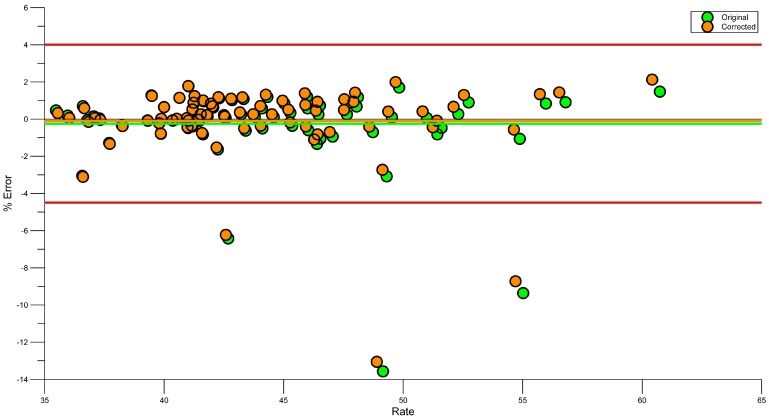
Bland Altman plot showing error in stroke rate after Regression correction. Green before correction, Orange after correction.

Equation (1) corrected the systematic bias from −0.25% to −0.1% (Figure 10), in line with previous research [23].

Stroke duration was defined as the time between consecutive hand entries. The accelerometer found the hand entry as the local LW_Y_ and RW_Y_ minima near the body being level. This identification of hand entry matched previous the findings [36,38], and was verified as the time from the initial synchronization ‘bumps’ to the hand entry on the video.

**Figure 11:**
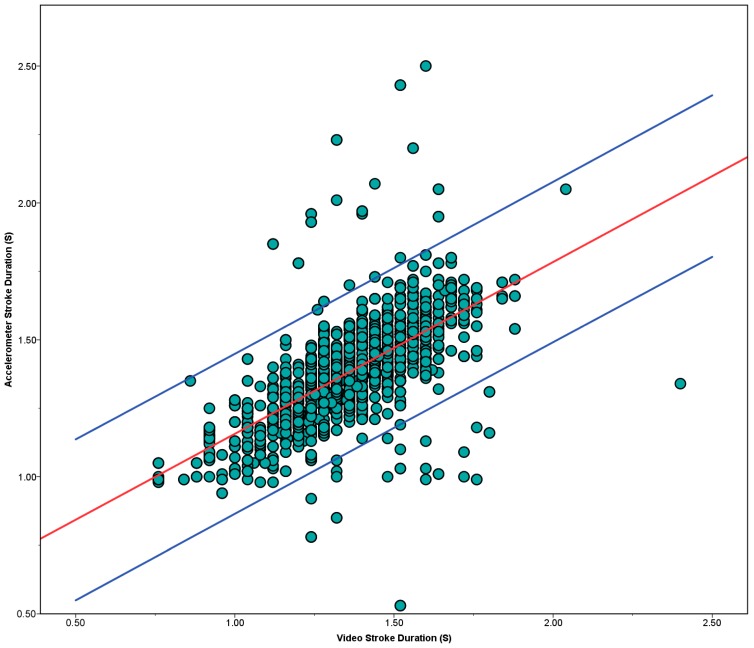
Stroke Duration Calculation. Black line shows correlation, red lines show 95% interval.

Stroke duration calculation between the video and accelerometers (n = 1028) shows a strong significant positive correlation, r = 0.637, *p* = 0.00. Whilst the results show a strong correlation, Figure 11 shows that there are a large number of outliers (outside 95% limits). Whilst hand entry time is visually definable by the ‘splash’; as the hand enters the water; and with these results being defined on a frame by frame basis, it can still be hard to define the precise point of entry. This is particularly true from the video angle of the global camera used. The video angle occasionally limited the accuracy of the hand entry by the kick splashes obscuring the view. This however, does demonstrate the strength of the sensors in their ability to correctly and consistently identify the hand entry points using the minima LW_Y_ and RW_Y_ axes.

Costill, Kovaleski, Porter, Kirwan, Fielding and King [62] and Chollet, Pelayo, Delaplace, Tourny and Sidney [63] have expressed that stroke length is the best indicator of performance. With the results presented here, the stroke length (distance per stroke) can be calculated. Bächlin and Tröster [20] developed from the stroke duration (SD) by incorporating *ῡ* to calculate the Stroke Length (Distance per Stroke—*DPS*) using Equation (2). Equation (2) Stroke Length Calculation (Method 1):

(2) $$SL=\bar{v}\cdot SD$$

Typically [64,65,66], stroke length is calculated as: (Equation (3) Stroke Length Calculation (Method 2)):

(3) $$SL=\frac{\bar{v}}{SR}$$

Whilst this method has been shown to overestimate distance per stroke by 4%–5% [67], it has been accepted as a systematic overestimation, which does not generally affect subsequent comparisons [65].

Outputs from both methods were compared (Equations (2) and (3)) and results showed that the method used by Bächlin and Tröster [20] produced stroke distances, on average, an additional 1.5% greater than that of the method used by Craig, Skehan, Pawelczyk and Boomer [64]. Perhaps Bächlin and Tröster [20] did not use this method, as their system did not calculate stroke rate. Because of this additional overestimation, the method utilized by Craig, Skehan, Pawelczyk and Boomer [64] was implemented in this system. As with Hawley, Williams, Vickovic and Handcock [65], no attempt was made to derive a correction factor for stroke distance.

### 3.4. Reliability of Phase Durations Detection

The algorithm developed was tested for reliability against the durations of the phases derived from the video. The video camera used for the underwater phases use a frame rate of 60 Hz, resulting in an image being taken every 0.0166 s. Results show (Table 3) that each phase has a relatively lower error. The Mean Absolute Error (MAE) of 0.06, 0.07, 0.06 and 0.08 s (respectively) also shows low error rates. There is little difference between the mean and MAE, which shows there is no major positive or negative difference, no positive or negative bias.

**Table 3:** Reliability statistics for Phase durations. All results presented in Seconds.

	Mean Error (±SD)	Mean Absolute Error (±SD)	95th Percentile for Absolute Error	Root Mean Squared Error	SEM	Systematic Bias	Random Error
Entry (n = 78)	0.00 (±0.08)	0.06 (±0.06)	0.15	0.08	0.06	0.00	0.16
Pull (n = 78)	0.04 (±0.10)	0.07 (±0.08)	0.20	0.10	0.07	−0.04	0.19
Push (n = 78)	0.03 (±0.07)	0.06 (±0.05)	0.15	0.08	0.05	0.03	0.15
Recovery (n = 71)	0.05 (±0.12)	0.08 (±0.1)	0.33	0.13	0.08	0.05	0.23

This error, in terms of an equivalent to video analysis, would relate to a difference between correct identification of a frame to within 3.6 frames (hand entry and push) to 4.8 frames (for recovery), which could easily be a disagreement between an analyst recording the phases from video.

It was found that although every effort was taken using frame-by-frame analysis to determine each phase, and using cooperation from the coach to aid in this at the start, there is still error in the analysis. This is shown with random error in the reliability ranging from 0.15 s to 0.23 s. Yeadon and King [68] note that in the synchronization of two video sources, there can be in induced error of 0.02 s. This error does not then include any disagreement between any observers made therein.

In terms of 2D video analysis the phases of the strokes were also found to be fairly subjective, with the swimmer making subtle movements during the pull and push which were at times directly towards the cameras and so were difficult to quantify the exact timings of each phase. There was also occlusion of the arms during the entry and exit of the arm from the water at times, meaning the error in the recovery phase was doubly penalized by the air-water interface. An error in the identification of the entry time also led to an additional error in the previous recovery phase.

**Figure 12:**
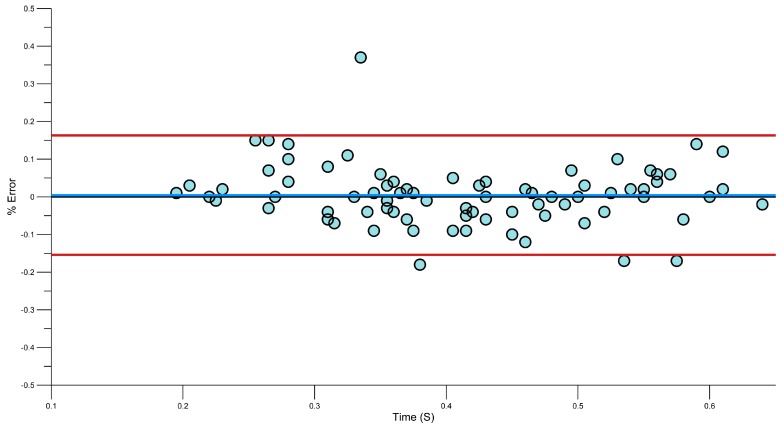
Bland-Altman plot of Entry Duration Error.

**Figure 13:**
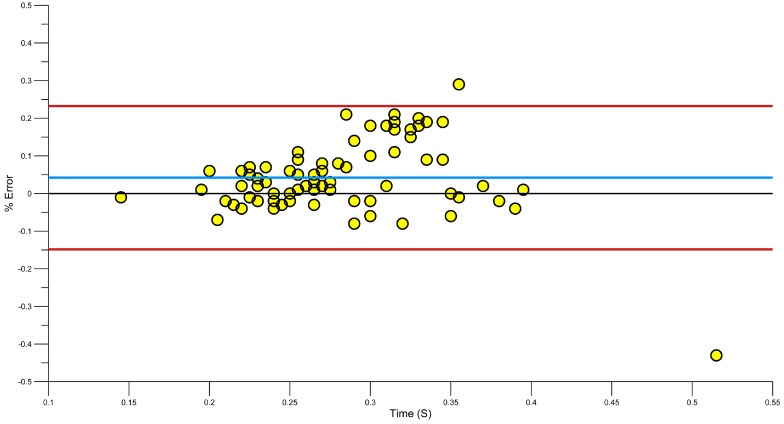
Bland-Altman plot of Pull Duration Error.

**Figure 14:**
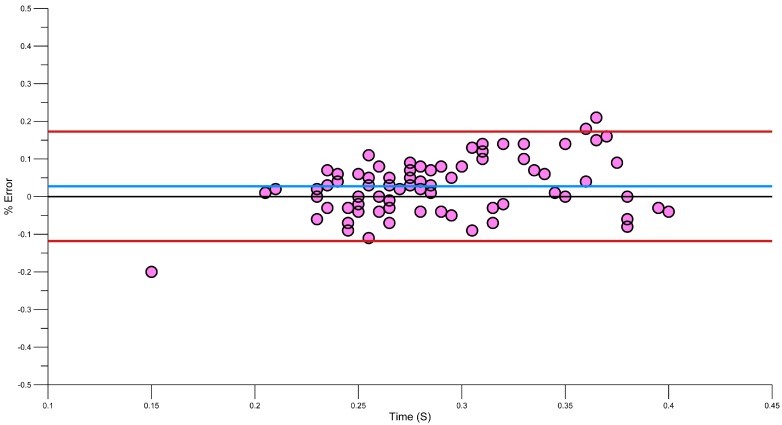
Bland-Altman plot of Push Duration Error.

**Figure 15:**
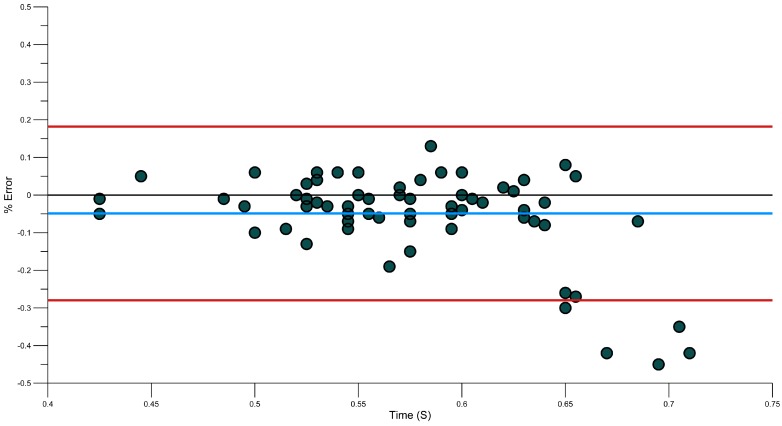
Bland-Altman plot of Recovery Duration Error.

Bland-Altman plots (Figure 12, Figure 13, Figure 14 and Figure 15) showing the relative error in percentage terms shows that for each phase there are only four entry phases which were outside of the 95% limits, two for the pull phase, three for push and five for the recovery. This shows some promising results.

### 3.5. Strengths

This study has extended on previous works by creating an algorithm using multiple sensors to determine the phases of the stroke. Whilst positive results have been shown for the phase detection and this algorithm worked well, there was some need for some personalization for some swimmers in this study. One method of over-coming this could be to use a percentage of the stroke time as a window to search through rather than hard-defined samples, a second would be visual confirmation of phases with a user input required. This could be built into a learning algorithm to reduce the need for human interaction in the future. This study has also determined that the arm sensor was of no use in refining the accuracy algorithm.

The swimming protocol used, with devices attached and removed, does demonstrate the test-retest ability of the placement of the devices on the body, in addition to the reliability of the systems processing capabilities.

### 3.6. Limitations

Due to the fatiguing set used (4 × 25 m with devices attached, 8 × 25 m (200 m) not measured, and 4 × 25 m (100 m) with devices attached), there were four laps, which required manual lap trimming, and four which were automatically trimmed. This increases the amount of human interaction required, however, a change in the protocol to increase the laps in the pre- and post-fatigue could reduce this.

### 3.7. Weakness 

The customization process in the algorithm entailed extending the ‘search window’ when identifying maxima and minima on the wrist sensor, relative to the body roll peak or zero crossing. Whilst this works, it does not offer a 100% full proof system for all swimmers.

### 3.8. Practical Applications for the Athlete and Coach

The fact that some customization was required highlights how each individual swimmer compensated in the technique in order to maintain as fast a lap time as possible. This agrees with the proposal by Davids, Glazier, Araújo and Bartlett [69] that individuals will adapt to perform the task to the best of their ability. This highlighted the importance of systems such as this, where multiple coordination changes are unlikely to be accurately diagnosed by the coach visually, reiterating Burkett and Mellifont [39] (p. 110) comment that,

“...*the demands of the swimming coach and athlete, objective data on the swim performance is required*”.

Performance-related feedback is one of the major factors, which contributes to an athlete’s development during the course of the motor skill acquisition process [70]. Most coaches will convey skill-based information to athletes through demonstrations and verbal instruction based on their, potentially, biased viewpoints. There is some concern that some coaches may tend to produce “one size fits all” assumptions [3]. Using a system such as this would allow coaches to develop tactical and technical training drills to help their swimmers, rather than them being based on pure supposition.

**Figure 16:**
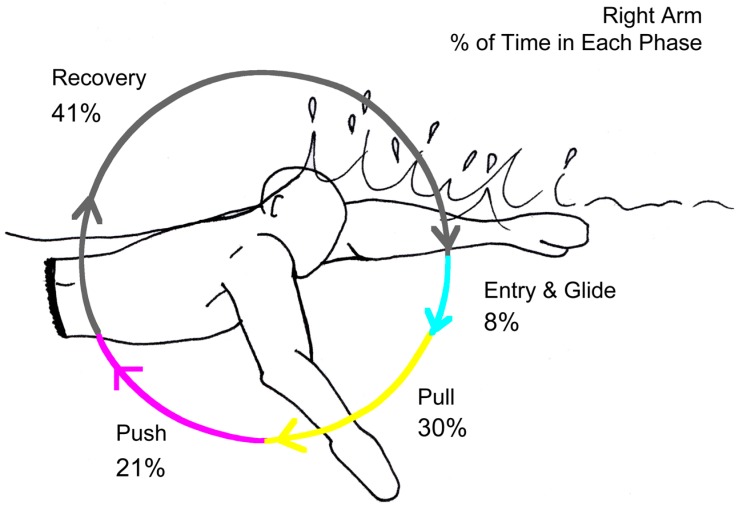
Example output of the system for coaches.

Most coaches will convey skill-based information to athletes through demonstrations and verbal instruction. During a sporting action, an athlete will gather intrinsic information using proprioceptive and exteroception sensory mechanisms, allowing them to rapidly adapt to the demands of the given task [2]. This is typically supplemented through augmented feedback (AF) [71], sometimes referred to as artificial feedback [2]. Extrinsic in nature and usually presented by a coach, AF can contain knowledge of results (KR) and/or knowledge of performance (KP). AF can be presented concurrently or terminally to the action. Where, KR will focus on information related to the outcome of a movement, KP will focus on the quality or pattern of the movement [71]. Performance-related feedback is one of the major factors, which contributes to an athlete’s development during the course of the motor skill acquisition process [70]. Systems that collect data, such as the one presented here, allow visualization of the data, which is an important part of the learning process [72]. Recording factors, such as the phases of the stroke, can allow the coach to identify what is going on under the water and make appropriate recommendations. An example of an output from the present system is shown in Figure 16 and can be used as extrinsic feedback. This is not to say that the complexities of swimming can be easily summarized in any one single image, but there needs to be some way of succinctly demonstrating to the coach what is happening to make coaching decisions. Future work should focus on developing visualization techniques for coaches, and testing these as also suggested by Rowlands, James and Lee [72].

## 4. Conclusions

This system demonstrates the capabilities of multiple sensor systems in processing multiple variables simultaneously on a swimmer. There are complex coordination structures in swimming, and with systems such as this, there is a strong possibility of finding, recording and developing visualization methods to demonstrate them. The developed algorithm uses a search window relative to the body roll (peak/trough) but required some customization. Four swimmers required individual adaptation to the stroke phase calculation method. This need to adapt some parts of the algorithm demonstrates that single sensor systems (such as sacrum mounted) will not be able to determine or infer the phases of the stroke with sufficient accuracy. Multiple sensor systems will be the future of monitoring in sport and require further development in terms of usability, visualization of output data and ease of synchronization.
